# Control of MAIT cell functions by cytokines in health and disease

**DOI:** 10.3389/fimmu.2025.1594712

**Published:** 2025-05-30

**Authors:** Laetitia Camard, Elisabetta Bianchi, Lars Rogge

**Affiliations:** Immunoregulation Unit, Department of Immunology, Institut Pasteur, Université Paris Cité, Paris, France

**Keywords:** MAIT cells, Cytokines, tissue repair, infectious diseases, inflammatory diseases, cell plasticity, MAIT cell activation, interleukins

## Abstract

Mucosal-associated invariant T (MAIT) cells are innate-like T cells that express a semi-invariant T cell receptor (TCR). These cells predominantly reside in tissues, such as the liver, lung, skin and the gastrointestinal tract. MAIT cells can be activated via their TCR that recognizes riboflavin metabolites presented by the MHC class I-related protein 1 (MR1). These cells can also be activated in a TCR-independent manner by cytokines, in particular IL-12 and IL-18, but also by type I interferons, IL-7, IL-15 and IL-23, underlining their innate-like characteristics. MAIT cells have important functions in antibacterial and viral immunity but also in tissue repair and homeostasis. Recent studies highlighted the plasticity of MAIT cells in response to cytokines, suggesting an important role of the cytokine milieu in modulating MAIT cell functions. Here, we discuss how cytokines control MAIT cell functions in various contexts.

## 1 Introduction

Mucosal-associated invariant T (MAIT) cells are a subset of innate-like T cells characterized by their semi-invariant T cell receptor (TCR) composed of *TRAV1–2* paired with *TRAJ33*, *TRAJ12* or *TRAJ20* in human and *Trav1* paired with *Traj33* in mouse; with a CDR3 region of constant length (1, 2). They are restricted by the MHC class I-related protein 1 (MR1) presenting metabolites derived from the riboflavin (vitamin B2) synthesis pathway, the most potent ligand being 5-OP-RU (5-(2-oxopropylideneamino)-6-D-ribitylaminouracil) (3, 4). In mammals, there is a striking conservation of MR1 and the *TRAV1–2* and *TRAJ33* genes (5), indicating strong selective pressure and suggesting that MAIT cells have important non-redundant functions.

MAIT cells are found in tissues at various frequencies. In humans, they represent in average 3% of CD3^+^ peripheral blood cells, are enriched in barrier tissues such as the digestive tract and the lungs, and are particularly abundant in the liver (6).

The MAIT cell phenotype has been extensively described. In humans, they are mainly CD8^+^ (~80%) and double negative (CD4^-^CD8^-^, ~15%) with very few CD4^+^ cells (7, 8), while murine MAIT cells are mostly double negative (9). Based on co-receptor expression patterns, some groups identified distinct MAIT cell subsets (10, 11), while others only detected minor differences and suggest that they rather belong to a continuum (12). MAIT cells have a CD45RA^-^CD45RO^+^CD95^high^CD62L^low^ effector memory phenotype. They express a specific pattern of chemokine receptors, with high levels of CCR6, CXCR6 and CCR5, intermediate levels of CCR9 and heterogenous expression of CXCR4, which endow their tissue tropism. MAIT cells also express some natural killer (NK) cell markers at heterogeneous levels including NKp30, NKp80, CD56, NKG2A and NKG2D (7, 13). The MAIT cell phenotype is also characterized by the expression of a broad range of cytokine receptors including interleukin 7 receptor (IL-7R), IL-18R, IL-12R, IL-15R and IL-23R (7, 13–17). MAIT cells are also defined by the expression of a specific set of transcription factors. They express the Promyelocytic Leukemia Zinc Finger protein (PLZF, encoded by *ZBTB16*), a critical transcription factor for the acquisition of innate-like functions (7, 18, 19). Human MAIT cells express high levels of retinoic-acid related orphan receptor gamma (RORγt) and intermediate levels of T-bet (encoded by *TBX21*), conferring them mixed type 1/17 phenotype and functions (7, 13, 20). In the mouse, MAIT cells are differentiated into MAIT1 and MAIT17 subsets, which express either T-bet or RORγt, respectively (21, 22).

MAIT cell activation can be achieved in a TCR-dependent manner, via the presentation of riboflavin metabolites on MR1 by antigen presenting cells. However, TCR signaling alone is not sufficient to induce full MAIT cell activation and requires additional signals that can be provided through co-stimulatory molecules such as CD28, or innate cytokines (23). Additionally, MAIT cells can be activated in a TCR-independent manner by cytokines, such as the combination of IL-12 and IL-18 (24). MAIT cell activation and localization are also modulated by other factors such as chemokines or prostaglandins (25, 26). In response to these signals, MAIT cells can perform a broad range of functions, including antimicrobial and antiviral defense, or tissue repair (27–29). Here, we review how cytokines modulate these various MAIT cell functions.

## 2 Role of cytokines in MAIT cell development

MAIT cell development is a three-stage process that has recently been reviewed elsewhere (30). Here, we will focus on the importance of cytokine signals at different stages of this process.

In the thymus, murine MAIT cells are selected by double positive thymocytes expressing MR1 and are initially CD24^+^CD44^-^ (stage 1). They differentiate into an immature stage 2 with CD24 downregulation and expression of CD62L. Stage 3 MAIT cells are CD24^-^CD44^+^, acquire PLZF expression and differentiate into MAIT1, expressing T-bet, and MAIT17, expressing RORγt, subsets (22, 30, 31). Similarly, human MAIT cell development stages are defined by the expression patterns of CD27 and CD161. Stage 1 MAIT cells are CD27^-^CD161^-^, differentiation into stage 2 is defined by the acquisition of CD27 expression, and stage 3 cells express CD161. Unlike in mice, human MAIT cells do not commit to type 1 or 17 lineages, but rather exhibit a homogeneous mixed 1/17 phenotype (13, 32–34).

In mouse, IL-18 is important for stage 2 to stage 3 transition as IL-18-deficient mice display reduced frequencies of stage 3 MAIT cells in the thymus and in peripheral tissues (31). Single cell transcriptomes and TCR repertoires analyses revealed that commitment to the MAIT1 or MAIT17 subsets is independent of TCR characteristics, suggesting the involvement of other signals which could likely be provided by cytokines (35). Supporting this model, a study identified IL-15 and IL-2 signaling through CD122 (IL-2Rβ) to be critical specifically for MAIT1 cell development and/or maintenance, while the co-stimulatory molecule ICOS was necessary for the MAIT17 subset development (36).

After thymus egress, murine MAIT cell subsets populate different tissues: MAIT1 preferentially colonize the spleen and liver; MAIT17 are mostly found at barrier sites including the lung, gut and skin (9, 37). The cues regulating this differential tissue colonization are not completely understood, yet there is some evidence that cytokine signals are involved. Indeed, IL-23R-deficient mice exhibit an impaired MAIT cell compartment in the skin (38). IL-23-deficient mice have reduced MAIT cell frequencies in the ileum and colon, and their type 1/17 tissue-specific phenotype is altered (33). Yet, MAIT cell numbers are normal in the lungs of *Il23a* ^-/-^ animals (39). Of note, MAIT cell numbers are also normal in the lungs of *Ifng* ^-/-^, *Il18* ^-/-^, *Il6* ^-/-^ and *Il12a* ^-/-^ mice under homeostatic conditions (39). Together, these studies point to a critical role for IL-23 signaling in the establishment of a MAIT cell population in the skin and in the gut but not in the lung, highlighting the importance of tissue-specific cues, including cytokines, in controlling MAIT cell tissue localization.

In human, inborn errors of immunity (IEI) - a heterogeneous group of diseases in which a germline variant causes defects in the immune system - provide invaluable insights into critical components of MAIT cell biology [reviewed in (40)]. A complete lack of MAIT cells has been observed in individuals with MR1 (41) or RORγt (42) deficiencies, highlighting that these proteins are essential for the development and/or maintenance of a MAIT cell population. In most IEI cases impacting the MAIT cell compartment, a reduced frequency of circulating MAIT cells is reported. These include variants in several cytokine receptors namely *IL12RB1*, *IL12RB2*, *IL21R*, *IL23R* and *IL6ST* (43–46). This suggests a critical role for these cytokines in the development and/or maintenance of human MAIT cells, yet the underlying mechanisms remain to be deciphered.

## 3 Tissue repair functions

In the past few years, tissue repair functions have been attributed to MAIT cells [reviewed in (47)]. Transcriptomic analyses of human and mouse MAIT cells have largely attributed the expression of this tissue repair program to TCR triggering (48–50). More recently in a mouse model of skin excision, du Halgouet et al. showed that MAIT cells exhibited a tissue repair program, were recruited to skin lesions and accelerated wound closure independently of TCR signaling. IL-18 was identified as an important inducer of amphiregulin production by MAIT cells, that was critical for their tissue repair functions (51). Thus, tissue repair functions of MAIT cells can be triggered by different modes of activation that could depend on the environment. More studies are required to fully understand the extent of MAIT cell tissue repair program, and its function in different contexts such as chronic inflammation versus acute lesion.

## 4 Fundamental studies on the effects of cytokines on MAIT cell functions

### 4.1 Combination of IL-12 and IL-18

The first studies identifying MAIT cell TCR-independent activation revealed that the combination of IL-12 and IL-18, that was previously shown to mediate NK cell activation (52), could potently activate MAIT cells and induce production of interferon γ (IFN-γ) (24, 53, 54). Further studies aimed at characterizing this TCR-independent response *in vitro*. TCR- and cytokine-mediated activation of MAIT cells have different kinetics and induce distinct responses. Cytokine-mediated activation peaks after 20–24 hours while TCR signaling is faster with the activation peak reached after 6 hours (24, 49). IL-12/IL-18 stimulation leads mainly to the production of IFN-γ, while TCR stimulation induces a more polyfunctional profile. The induced cytotoxic profiles are also different with increased production of granzymes A, K and M by cytokine-activated MAIT cells (49). Additionally, although activation of MAIT cells results in a core activated transcriptomic signature, the two modes of activation lead to distinct transcriptomic profiles. Finally, it is noteworthy that there is a synergy between the two modes of activation, that results in enhanced effector functions and a specific transcriptomic profile (49, 50).

Further studies have aimed at deciphering specific mechanisms underlying IL-12 and IL-18-mediated MAIT cell activation. Specifically, it has been demonstrated that IL-12 and IL-18 synergize together and with TCR signaling for optimal IL-17 production (55). IL-15 and tumor necrosis factor (TNF)-like protein 1A (TL1A) also enhanced effector functions of human blood and gut MAIT cells stimulated *in vitro* with suboptimal concentrations of IL-12 and IL-18 (50). A cocktail of inflammatory cytokines containing IL-12, IL-18 and IL-15 promoted sustained CTLA-4 expression on MAIT cells (56).

### 4.2 Individual cytokines

**Figure 1:**
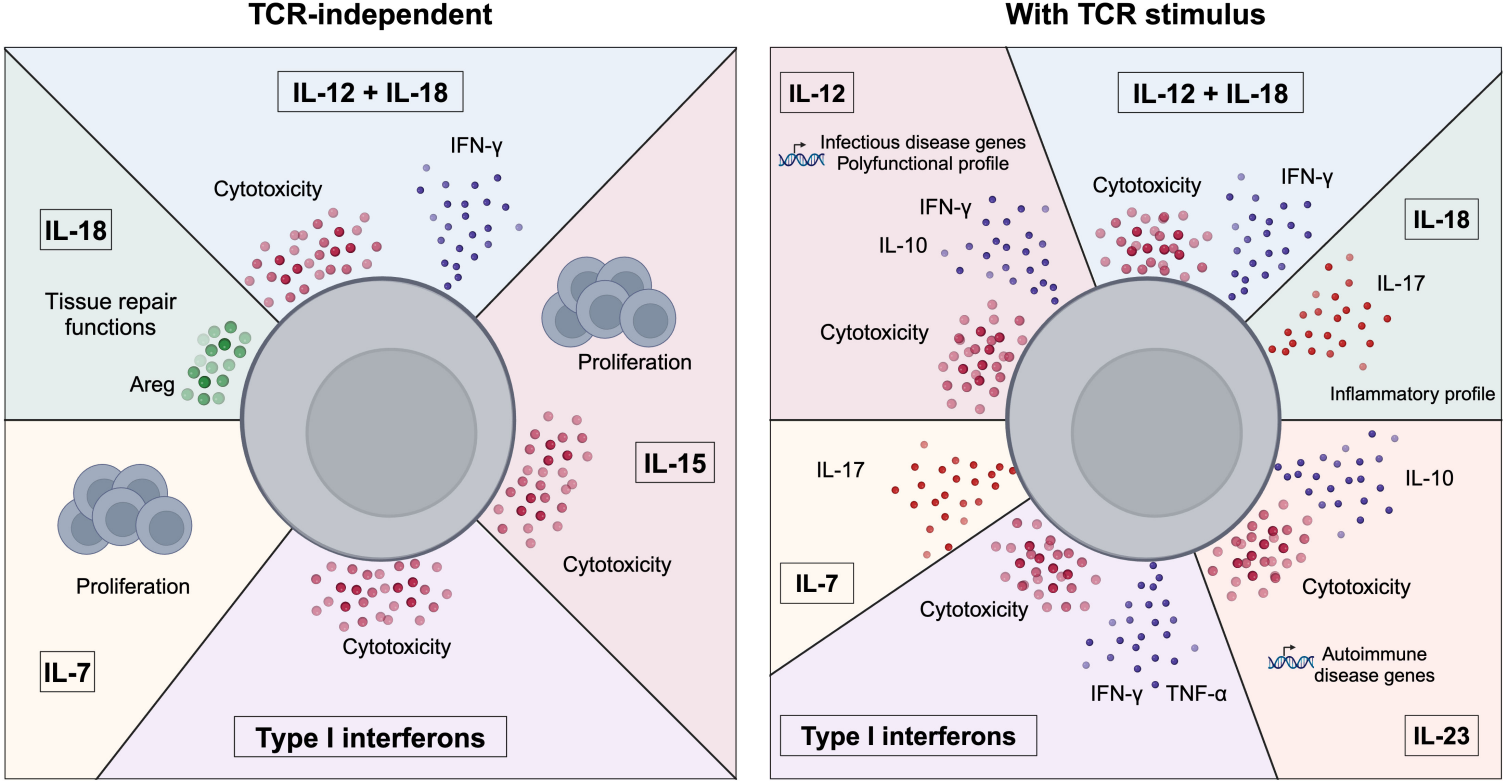
Control of MAIT cell functions by cytokines. Summary of the effects of cytokines on MAIT cell functions. MAIT cells can be activated in the absence of a TCR stimulus (left panel) by cytokines such as IL-7, IL-18, IL-15, type I interferons or the combination of IL-12+IL-18. MAIT cells exhibit various responses to cytokines as they can secrete pro-inflammatory cytokines (e.g. IFN-γ), cytotoxic mediators or adopt a tissue repair program. The cytokine milieu can also influence MAIT cell functions in the presence of a TCR stimulus (right panel). MAIT cells can adopt distinct functional and transcriptional profiles in response to different cytokines, highlighting their plasticity.

Besides IL-12R and IL-18R, MAIT cells express a broad range of cytokine receptors that prompted the analysis of the effects of other cytokines on their activation. The effects of these cytokines on MAIT cell responses are summarized in **Figure 1**. Specifically, MAIT cells express very high levels of IL-7R (14). IL-7 stimulation induced proliferation of peripheral CD161^high^IL-18Rα^+^CD8^+^ T cells and liver-derived MAIT cells (57, 58). Several studies identified IL-7 as an important cytokine promoting IL-17A production by MAIT cells. Indeed, priming of human peripheral and liver MAIT cells with IL-7 before TCR stimulation resulted in enhanced MAIT cell activation and secretion of IFN-γ and IL-17A, while priming with the classical type 17 inducing cytokines IL-1β and IL-23 mostly induced IFN-γ production (14). Similarly, priming of peripheral MAIT cells from axial spondyloarthritis patients with IL-7 but not with IL-23 increased activation and IL-17A production (59). A study on MAIT cells from the salivary glands of primary Sjogren’s Syndrome patients revealed that IL-17A production was induced *in vitro* by two different pathways. IL-7 induced *IL17A* concomitantly with *STAT3*, *HIF1A* and a decrease of *RORC*, while IL-23 increased *IL17A* expression together with the master type 17 transcription factor *RORC* (60). Additionally, IL-7 stimulation with a low dose of fixed *E. coli* induced production of IL-17A, IFN-γ and cytotoxic mediators by MAIT cells (20).

Another important cytokine in MAIT cell biology is IL-15. Similarly to IL-7, stimulation with IL-15 alone induced proliferation of peripheral and liver-derived MAIT cells (57, 58). In liver-derived MAIT cells, IL-15 induced the highest cytotoxic killing capabilities compared to IL-2, IL-7 and IL-12 which induced modest killing. This mechanism is TCR-independent and mediated by NKG2D and granzyme B (58). IL-15 stimulation of peripheral blood mononuclear cells (PBMC) also induced cytotoxicity in MAIT cells, accompanied by IFN-γ production, through a MR1-independent mechanism and mediated by IL-18, mainly secreted by monocytes in this context. However, adding IL-18 alone to PBMC cultures was not sufficient to recapitulate MAIT cell activation induced by IL-15 and trigger IFN-γ production, highlighting the importance of the integration of multiple signals to control MAIT cell effector functions (61). In a co-culture model of MAIT cells with mesenchymal stem cells, MAIT cell activation was enhanced by enhancing autophagy in these cells via IL-15 and independently of MR1 and cell-cell contact (62).

Type I interferons, mainly IFN-α and IFN-β, are important activators of MAIT cells. Lamichhane et al. revealed that type I interferons alone were able to activate cytotoxic responses of MAIT cells, and combined with TCR stimulation additionally enhanced effector cytokine secretion (63). Type I interferons are also important in mediating the effects of toll like receptor (TLR) 7 and 8 signaling in PBMC cultures. In particular, IFN-α enhanced MAIT cell response to anti-CD3 stimulation only when added first, indicating a priming role for type I interferons rather than a co-activator role per se (64).

## 5 Cytokines drive functional MAIT cell plasticity

Although human (as well as sheep, cattle, opossum and bat) MAIT cells are a rather homogeneous population (33, 65), they exhibit a broad spectrum of functions, suggesting that their responses can be adapted to their environment. Bulk transcriptional analyses revealed that TCR and cytokine stimulations elicited distinct transcriptional programs in MAIT cells (49, 50). This was further validated using single cell transcriptomics (12). Specifically, the transcriptional responses to TCR or IL-12/IL-18 stimulation followed distinct activation trajectories. Analysis of changes in transcription factor activity identified specific regulons for each of the two modes of activation, for example STAT1 and IKZF1 were specific for cytokine-mediated activation. Stimulation with both TCR and cytokines induced an IL-17-expressing population, that was not detected with either of the single stimulation, highlighting the synergy of these signals. Except for the expression of IL-17, this population was not transcriptionally, or clonally different from the non-expressing ones, suggesting that they do not represent a distinct subset of cells but result from functional plasticity (12). We and others also profiled MAIT cell responses to various cytokines in the presence of a TCR stimulus and identified different functional polarizations (17, 66). IL-23 enhanced IL-10 production, cytotoxicity and expression of autoimmune-related genes, mediated by the AP-1 family member Basic Leucine Zipper ATF-Like Transcription Factor (BATF). IL-18 polarized MAIT cells to an inflammatory profile, and drove IL-17 production (66) while IL-12 induced a diverse profile, including immunoregulatory mediators such as IL-10 and infectious disease related genes (17, 66). IL-12 induced IL-10 secretion in a c-Maf-dependent manner (66). These studies underline the functional and transcriptional adaptability of MAIT cells to the cytokine environment, and their capability to adopt not only inflammatory profiles but also to mediate anti-inflammatory responses.

A report in the mouse also highlighted functional plasticity of MAIT cells. In this model, MAIT17 cells can convert into functional MAIT1 cells that protected mice against bacterial infections (67). Altogether, the cytokine environment is important in driving MAIT cell plastic responses.

## 6 Cytokines modulate antibacterial activity of MAIT cells

The most appreciated role of MAIT cell is their antibacterial activity, mediated by the recognition of bacteria through MR1 presenting 5-OP-RU derived from bacteria (4, 27). However, the antibacterial responses do not entirely rely on antigen-dependent activation of MAIT cells and involve additional signals that can be provided by cytokines.

Several studies identified IL-12 and IL-18 as key cytokines mediating MAIT cell antibacterial activity by promoting IFN-γ production and cytotoxicity. These interleukins are important for *in vitro* MAIT cell responses to various bacteria including Bacillus Calmette-Guérin (BCG, 59, 60), *M. tuberculosis* (70), *S. pneumoniae* (71), *C. difficile* (72), *F. tularensis* (73) or *S. aureus* (74). It is noteworthy that the need for additional TCR signal depends on the context since in response to BCG one study on mouse cells described a MR1-independent mechanism (68), while another one using human PBMC revealed a synergy of TCR and cytokine signaling (69). Interestingly, the relative importance of TCR or cytokine signaling for MAIT cell activation was dependent on the antigen presenting cell (71). Further highlighting the importance of the environment in controlling MAIT cell functions, a study investigating the role of IL-18 in *Francisella* infections demonstrated that this cytokine was critical for *in vitro* production of IFN-γ by MAIT cells, but was dispensable *in vivo* (75). IL-12 and IL-23 were crucial in promoting MAIT cell responses *in vivo* upon *F. tularensis* infection as mice lacking either one of these cytokines exhibited impaired MAIT1 cell numbers in the lung. It is noteworthy that *Il12p40* ^-/-^ mice, lacking both cytokines, had a more severe defect than *Il12p35* ^-/-^ or *Il23p19* ^-/-^ deficient in IL-12 or IL-23 respectively; pointing to nonredundant or synergistic functions of these interleukins in MAIT1 cell responses (76). Administration of IL-12 during *L. longbeachae* (MAIT17-polarizing) infection resulted in dose-dependent increased MAIT1 responses, highlighting the modulation of MAIT cell responses to bacterial infection by the cytokine milieu (76). IL-12 was also shown to synergize with IL-7 secreted by macrophages and MR1 signaling upon *Nontypeable Haemophilius influenzae* infection, to induce granzyme B production by MAIT cells (77).

Furthermore, type I interferons have been shown to be important to enhance MAIT cell effector functions in response to *E. coli in vitro* (63). In *K. pneumoniae* infection in the mouse, MAIT cells were activated by type I interferon, produced IFN-γ and granzyme B and had a protective role. In human MAIT cells, transcriptomic analyses revealed that *K. pneumoniae* induced a type I interferon signature; and MAIT cell activation was dependent on type I interferon. Interestingly, *K. pneumoniae*-mediated activation of both murine and human MAIT cells was MR1-independent, even though this bacteria possesses the riboflavin pathway (78). Other studies also highlighted the role of IL-15 and TNF-α in enhancing MAIT cell effector responses to *M. tuberculosis* and *E. coli* challenges respectively (79, 80). Additionally, IL-23 was important for MAIT cell activation and accumulation in the lungs in mice infected with *S. typhimurium*, and vaccination using a combination of 5-OP-RU and IL-23 induced protection against *L. longbeachae* (39).

Altogether, various cytokines can modulate antibacterial activity of MAIT cells. The effects of cytokines on MAIT cells in response to bacteria are summarized in **Table 1**. Remarkably, there is a complex integration of antigen and cytokine signals that depend on the context and allow fine tuning of MAIT cell responses.

**Table 1:** Cytokines modulate antibacterial activity of MAIT cells.

Bacteria	Cytokine	Effect on MAIT cells	TCR dependency	Model	Reference
*BCG*	IL-12p40	IFN-γ production, inhibition of BCG growth in infected macrophages	Independent	*In vitro* (mouse cells)	(68)
*BCG*	IL-12 and IL-18	IFN-γ production	Addition	*In vitro* (human PBMC)	(69)
*M. tuberculosis*	IL-2, IL-12 and IL-18	IFN-γ and GzmB production	Independent	*In vitro* (human PBMC)	(70)
*S. pneumoniae*	IL-12 and IL-18	Activation (CD69 upregulation) and IFN-γ production	Independent with monocytes; addition with macrophages	*In vitro* (human PBMC)	(71)
*C. difficile*	IL-12 and IL-18	Activation (CD69 upregulation), IFN-γ production and cytotoxicity	Addition	*In vitro* (human PBMC)	(72)
*S. aureus*	IL-12	IFN-γ production and cytotoxicity, killing of infected cells and inhibition of intracellular persistence of *S. aureus*	Addition	*In vitro* (human PBMC)	(74)
*F. tularensis*	IL-12p40	Production of IFN-γ, TNF-α and IL-17A, control of *F.tularensis* intracellular growth	Addition	*In vitro* (mouse)	(73)
*F. tularensis*	IL-18	IFN-γ production	Independent	*In vitro* (mouse)	(75)
*M. tuberculosis*	IL-15	Enhanced IFN-γ production	Addition	*In vitro* (human PBMC)	(79)
*E. coli*	Type I interferons	Enhanced production of IFN-γ and TNF-α and cytotoxicity	Addition	*In vitro* (human PBMC)	(63)
*K. pneumoniae*	Type I interferons	Activation, IFN-γ and GzmB production	Independent	*In vivo* (mouse)	(78)
*K. pneumoniae*	Type I interferons	Activation, type I interferon transcriptomic signature	Independent	*In vitro* (human PBMC)	(78)
*E. coli*	TNF	Enhanced IFN-γ production	Addition, and with IL-12/IL-18	*In vitro* (human PBMC)	(80)
*S. typhimurium*	IL-23	Accumulation in the lungs and activation	Independent	*In vivo* (mouse)	(39)
*L. longbeachae*	IL-23	Vaccination induced protection against bacterial challenge	Addition	*In vivo* (mouse)	(39)
*Nontypeable heamophilus influenzae*	IL-12 and IL-7	GzmB production	Addition	*In vitro* (human PBMC)	(77)

Table summarizing the effects of cytokines on MAIT cell effector functions in response to various bacteria. The TCR dependency column indicates whether the described effects of the cytokines are TCR-independent or synergize with TCR signals (addition). The model column specifies if the studies were performed *in vitro* or *in vivo*, and in human or mouse.

## 7 Cytokines drive MAIT cell responses to viral infections

Albeit initial studies suggested that MAIT cells could not respond to viral infections but were rather antibacterial (27, 81), it is now clear that MAIT cells can be activated by viruses through cytokines, in a TCR-independent manner. **Table 2** summarizes the effects of cytokines on MAIT cells in response to various viruses.

**Table 2:** Cytokines drive MAIT cell responses to viral infections.

Virus	Cytokine	Effect on MAIT cells	Model	Reference
Dengue virus	IL-12, IL-18 and type I IFN	Activation (CD69 upregulation) IFN-γ, TNF-α and GzmB production	*In vitro* (human PBMC)	(28)
Influenza A virus	IL-18 and type I IFN			
Hepatitis C virus	IL-18, IL-15 and type I IFN			
Influenza A virus	IL-18	Activation (CD69 upregulation) IFN-γ and GzmB production	*In vitro* (human PBMC + A549 human lung epithelial cells)	(82)
Influenza A virus	IL-18	Recruitment	*In vivo* (mouse)	(83)
	IL-12, IL-18, IL-15 and type I IFN	CD25 upregulation, IFN-γ production Protective role of MAIT cells		
Epstein-Barr virus	IL-18	GzmB and IFN-γ production	*In vitro* (human PBMC)	(93)
Zika virus	IL-12 and IL-18	IFN-γ production	*In vitro* (human PBMC)	(94)

Table summarizing the effects of cytokines on MAIT cell phenotype and effector functions in response to the indicated viruses. The model column specifies if the studies were performed *in vitro* or *in vivo*, and in human or mouse.

Van Wilgenburg et al. showed that human MAIT cells are activated *in vitro* by antigen presenting cells infected with various viruses including dengue virus, influenza or hepatitis C virus. Viral activation of MAIT cells was not dependent on TCR signaling, but mediated by cytokines (IL-18, IL-15 and type I interferons) with a central role for IL-18. Importantly, the activation required more than one cytokine, and the ones involved depended on the virus (28). Furthermore, co-culture of PBMC with lung epithelial cells infected with influenza A virus (IAV) elicited MAIT cell activation, which was mediated by IL-18 and required CD14^+^ monocytes (82). Upon IAV infection *in vivo*, MAIT cell recruitment was impaired by IL-18 deficiency, while their activation was affected by deficiency of IL-15, IL-18, IFNαR and most dramatically of IL-12; indicating the involvement of various cytokines in coordinating MAIT cell responses to viral infections. In this context, MAIT cells protected against lethal influenza infection, at least in part through production of IFN-γ (83). In a mouse model of bleomycin-induced sterile lung injury, MAIT cells were similarly recruited and activated by IFN-α and IL-18 and had protective functions (84).

Further studies have demonstrated the antiviral functions of MAIT cells against other viruses. Notably, MAIT cells were shown to decline in the circulation of patients with active Covid-19 disease, and exhibited a strongly activated phenotype (85–88). The MAIT cell phenotype correlated with disease severity and IL-18 plasma concentration, and the authors identified a monocyte/macrophage shift from IFN-α to IL-18 production. This suggested that MAIT cell functions were altered by the pro-inflammatory environment, in particular IL-18, and may contribute to disease severity (86). Another study identified defects in MAIT cell responses from Covid-19 patients and proposed a mechanism by which IFN-α triggered important IL-10 production by suppressive monocytes, which impaired MAIT cell responses (88). Altered responses to IL-12/IL-18 stimulation were observed *in vitro* with MAIT cells from Covid-19 patients, and were partially rescued by IL-7 stimulation (87).

In human immunodeficiency virus (HIV) infections, several studies identified reduced frequencies and impaired responses of MAIT cells in the peripheral blood of patients (20, 89–91). IL-7 was important in arming MAIT cells with cytotoxic capabilities that were defective in HIV patients (20), and IL-7 treatment restored MAIT cell frequency in HIV-infected patients (92). Variants in *IL7RA* were also associated with the frequency and functionality of MAIT cells in HIV patients (90). A study suggested a mechanism to explain impaired MAIT cell functionality in HIV-infected patients: sustained type I interferon signaling induced IL-10 production by monocytes, and reduced IL-12 that together reduced MAIT cell antibacterial responsiveness (89).

MAIT cells also exhibited decreased frequency and activated phenotype in the blood of patients with Epstein-Barr virus (EBV)-associated T/NK lymphoprolipherative disorder, in correlation with disease severity and IL-18 plasma concentration. *In vitro* infection of PBMC with EBV induced IL-18 secretion by monocytes, and blocking this cytokine reduced MAIT cell activation suggesting that IL-18 may be important in MAIT cell phenotype in this disease (93). Similarly, in dengue infected patients, MAIT cells had an activated phenotype, and their activation *in vitro* by Zika virus was blocked when antibodies blocking IL-12 and IL-18 were added to the culture (94).

## 8 MAIT cells in inflammatory diseases

It has been reported in many inflammatory diseases, such as inflammatory bowel disease (IBD, 85, 86), multiple sclerosis (97), type 1 diabetes (98) or primary Sjogren’s Syndrome (60, 99), that MAIT cell frequency is reduced in the periphery [reviewed in (100)]. Given the chemokine receptor pattern expressed by MAIT cells, it was suggested that these cells were recruited to the inflamed tissues. Supporting this hypothesis, MAIT cells are reduced in the blood and enriched in the inflamed mucosa of IBD patients (95, 96). The plasma levels of pro-inflammatory cytokines such as IL-18 have been correlated with MAIT cell frequencies and phenotype (97). These observations together with the known responsiveness of MAIT cells to cytokine stimuli suggest that they could have a role in these diseases. However, to our knowledge, no mechanism of MAIT cell activation by cytokines in the context of inflammatory diseases has been described to date. The precise functions of MAIT cells in inflammatory diseases and the underlying mechanisms remain to be deciphered.

## 9 Discussion

Cytokines are key modulators of MAIT cell functions, as they can drive MAIT cell activation in the absence of TCR signaling in an innate-like manner. Since they modulate or synergize with the effects of TCR signaling, cytokines are also important in MAIT cell responses to antigen recognition. Many reports have focused on IL-12 and IL-18 to identify the TCR-independent activation programs of MAIT cells, or in the context of bacterial and viral infections. Yet, MAIT cells express a broad range of cytokine receptors, rendering them responsive to many other signals that remain underexplored. For example, IEI suggest important roles for IL-21 in human MAIT cell biology (46). However, to our knowledge, the effects of IL-21 on MAIT cell have not been assessed.

Furthermore, many studies highlight the importance of the context and the integration of multiple signals to fully activate MAIT cells and fine tune their functions. Thus, it would be of interest to further explore how different combinations of cytokines, other than IL-12/IL-18, in the presence or absence of TCR stimulus, can regulate the functions of MAIT cells. Additionally, further work is required to decipher how cytokine signals are integrated with various cues such as chemokines, interactions with other cells or the microbiota. These are indeed important for MAIT cells to adopt their broad effector functions in tissue-specific contexts.

There is an increasing number of reports denoting functional plasticity of MAIT cells, driven by the cytokine milieu. This sensing of the microenvironment enables MAIT cells to finely adapt their functions, either pro- or anti-inflammatory, to their tissue localization and to the homeostatic or inflammatory contexts. More work is required to decipher the underlying mechanisms, including the transcriptional networks and epigenetic processes possibly involved.
